# Does the Japanese insurance system increase outpatient psychiatric treatment for children and adolescents? A retrospective study using open data from the national claims database for 2016 to 2022

**DOI:** 10.1002/pcn5.70131

**Published:** 2025-06-11

**Authors:** Masahide Usami, Yoshinori Sasaki, Masahiro Ishida, Saori Inoue, Masaya Ito, Katsunaka Mikami, Noa Tsujii, Naoko Satake

**Affiliations:** ^1^ Department of Child and Adolescent Psychiatry, National Kohnodai Medical Center Japan Institute for Health Security Chiba Japan; ^2^ Department of Psychiatry and Behavioral Sciences Institute of Science Tokyo Graduate School Tokyo Japan; ^3^ Department of Psychiatry Fukuoka University Fukuoka Japan; ^4^ Department of Child Psychiatry Ehime University Graduate School of Medicine Toon City Japan; ^5^ National Center for Cognitive Behavior Therapy and Research National Center of Neurology and Psychiatry Kodaira Japan; ^6^ Department of Psychiatry Tokai University School of Medicine Kanagawa Japan; ^7^ Department of Child Mental Health and Development Toyama University Hospital Toyama Japan; ^8^ Department of Psychiatry, National Kohnodai Medical Center Japan Institute for Health Security Chiba Japan

**Keywords:** child, health insurance, mental health

## Abstract

**Aim:**

The prevalence of mental health issues among children and adolescents in Japan is rising, including school refusal, suicide, and neurodevelopmental disorders. In response, the Ministry of Health, Labour, and Welfare (MHLW) introduced insurance incentives in 2014 to expand access to outpatient psychiatric services for Japanese youth.

**Methods:**

This retrospective cohort study analyzed data from the National Database of Health Insurance Claims and Specific Health Checkups of Japan from fiscal years 2016 to 2022. Outpatient psychotherapy claims under the newly introduced insurance categories were analyzed by year, age group, sex, and prefecture. National trends and regional disparities were assessed using linear regression models.

**Results:**

Nationwide outpatient psychotherapy claims nearly doubled, from 521.0 per 10,000 youth in fiscal year 2015 to 1034.5 in fiscal year 2022 (*p* < 0.001). The increase was most pronounced among adolescent girls. By fiscal year 2022, 94% of prefectures had implemented the policy. Some regions, such as Tokushima and Yamanashi, experienced more than a threefold increase. Nevertheless, regional disparities between prefectures remained.

**Conclusion:**

The MHLW's insurance reforms significantly increased access to psychiatric care for children and adolescents across Japan. The combined impact of national financial incentives and local medical subsidies likely contributed to increased service utilization, given the reduction in additional costs for families. Ongoing policy efforts are needed to ensure equitable access and high‐quality care nationwide.

## INTRODUCTION

Japan's healthcare system plays a critical role in supporting the mental health of children and adolescents. Emerging challenges in recent years, such as school refusal, suicide, and rising diagnoses of neurodevelopmental disorders, indicate the need for greater access to psychiatric services.^1^, ^2^

For instance, in fiscal year (FY) 2024, the rate of school absenteeism among elementary and junior high school students continued to increase.^2^, ^3^ Suicide among individuals under 18 years of age reached 524 cases in 2024, further highlighting a critical public health concern. Furthermore, diagnoses of autism spectrum disorder and attention‐deficit/hyperactivity disorder, which require timely psychiatric intervention, have been increasing sharply.^4^, ^5^

Despite this growing need, Japan's current psychiatric care system remains significantly limited. A 2017 government report indicated that waiting times for initial consultations for developmental disorders frequently exceeded 3 months, with some institutions reporting waitlists of over 300 individuals.^6^ This inadequacy is further compounded by the lack of recognition of child and adolescent psychiatry as an independent specialty, which hinders the training and, thus, the availability of specialists—particularly in rural areas.^7^, ^8^

In response, the Ministry of Health, Labour, and Welfare (MHLW) introduced a series of reimbursement reforms targeting psychiatric care for youth. Since 2012, an additional fee for outpatient psychotherapy for patients under 20 years has been implemented. Although reforms began in 2012, limitations of NDB Open Data restrict our analysis to FY2014–2022, so earlier policy effects are not captured in this study. From 2016 to 2021, three additional reimbursement categories were gradually implemented. These included additional insurance points for outpatient psychotherapy for individuals under 16 and under 20 years of age.

This study evaluated whether these insurance policy changes increased the utilization of outpatient psychiatric services for children and adolescents. Using aggregated national claims data spanning 7 fiscal years, we assessed trends by age, sex, and region to establish the policy's impact. However, outpatient psychiatric claims for children and adolescents in Japan do not include mental health services from pediatric clinics, developmental consultations, or general practices.

Thus, this study contributes to the growing field of evidence‐based policymaking in psychiatry and underscores the potential of strategic reimbursement reform as a transformative tool for mental health system development in Japan and beyond.

## METHODS

### Japan's healthcare system

Japan implements a universal health insurance system. To address increasing concerns over developmental disorders, school absenteeism, and suicide among youth, the MHLW revised reimbursement policies to improve access to psychiatric care for children and adolescents.

Since 2012, an “additional fee for outpatient psychotherapy (under 20 years)” has been implemented. From 2016 to 2021, the following reimbursement categories were gradually implemented:

1. Additional fee for outpatient psychotherapy (under 20 years)
2. Additional fee for specialized management of child and adolescent psychiatry (under 16 years)
3. Additional fee for the specialized management of child and adolescent psychiatry (under 20 years).

Claims for the specialized management fees under Category 2 only applied to institutions that met specific criteria, while others could claim only the general outpatient psychotherapy fee under Category 1. Notably, the “under 16” management fee could be billed only once per institution.

In 2022, the system was further stratified into four categories, thereby expanding eligibility based on age and treatment duration. Certification by the Japanese Society of Child and Adolescent Psychiatry was not required. However, because specialist certification data are unavailable from the claims database, this study could not evaluate provider qualifications. However, these stringent requirements ensure that the specialized management fee functions as a built‐in clinical quality‐assurance mechanism: by mandating care be delivered by board‐certified child and adolescent psychiatrists (or under their direct supervision), requiring comprehensive, evidence‐based assessments and 60‐min therapeutic sessions for complex cases, and stipulating appropriate facility standards, the fee schedule guarantees that only providers meeting high clinical and infrastructure benchmarks can claim the enhanced reimbursement.

General outpatient psychotherapy costs around 3000 yen, while additional fees range from 3000 to 12,000 yen. In most areas, local governments subsidize care for those under 18, minimizing out‐of‐pocket costs.

### Data source

This retrospective cohort study analyzed data from the National Database of Health Insurance Claims and Specific Health Checkups of Japan (NDB Open Data) published by the MHLW, which provides comprehensive, anonymized, aggregated national insurance claims data. The NDB is publicly accessible to all researchers.^9^

The dataset encompasses inpatient and outpatient services by sex, age group, region, and service year. Japan's fiscal year runs from April to March. Data from FY2014 to FY2022 were analyzed.

### Study population and outcome measures

We extracted the annual claims for outpatient psychotherapy with additional fees from FY2016 to FY2022. This study could use distinct codes for psychotherapy sessions lasting <30 min versus ≥30 min. However, session‐level duration data were not available in the aggregated dataset. Population estimates by prefecture were obtained from the Basic Resident Register^10^ to determine per capita claim rates.

### Statistical analysis

Trends were analyzed using linear regression models (ordinary least squares) implemented in PRISM for Mac (GraphPad Software, Boston, USA). National trends were evaluated based on outpatient psychotherapy claims per 100,000 youth. Regional disparities among prefectures were assessed using the minimum to maximum ratio of outpatient psychotherapy claims per 10,000 youth under age 19 years. Subgroup analyses by age and sex included annual effect size calculations. A *p*‐value < 0.05 was considered statistically significant.

### Ethics

The NDB Open Data are publicly available and anonymized. No ethical approval or patient consent was required under the Japanese guidelines.

## RESULTS

### Declining birthrate in Japan

According to the national census data, Japan's total population decreased from 127.10 million in 2014 to 122.49 million in 2022. The population under 19 years of age fell from 22.54 million in FY2015 to 20.65 million in FY2022, a reduction of 1.89 million, which clearly demonstrates Japan's ongoing demographic decline.

### Trends in outpatient psychotherapy claims (all ages/0–19 years)

The total outpatient psychotherapy claims (including general psychotherapy, cognitive‐behavioral therapy [CBT], and group therapy) increased from 59.5 million in FY2014 to 69.4 million in FY2022—a 16.5% increase (Table 1). This growth likely reflects the rising demand, increased public awareness, and expanded access to evidence‐based psychiatric services.

**Table 1:** Trends of the number of claims in outpatient psychotherapy claims (all ages/0–19 years).

	0–19 years		All ages
FY2015	**2,604,906**	4.30%	61,191,289
FY2016	**2,810,594**	4.50%	61,839,713
FY2017	**3,054,238**	4.80%	63,418,056
FY2018	**3,269,494**	5.00%	64,805,124
FY2019	**3,461,649**	5.20%	66,198,805
FY2020	**3,614,392**	5.60%	64,549,221
FY2021	**4,170,348**	6.10%	68,141,614
FY2022	**4,365,557**	6.30%	69,439,156

Among individuals aged 0–19 years, claims increased from 2,604,906 in FY2015 to 4,365,557 in FY2022 (+67.6%). The most notable year‐on‐year increase occurred between FY2020 and FY2021, during the COVID‐19 pandemic.

### Trends in claims for additional fees fr the specialized management of child and adolescent psychiatry (FY2016–FY2022)

Outpatient psychotherapy claims under the new reimbursement categories increased from 631,855 in FY2015 to 1,061,284 in FY2022, indicating increased uptake of specialized management fees (Table 2).

**Table 2:** Additional fees for the specialized management of child and adolescent psychiatry.

	Additional fee for specialized management of child and adolescent psychiatry			Additional fee for child and adolescent psychiatry	Total
	<16 years			<20 years	
	>2 years	<2 years	<20 years, >1 hour/<3 months		
**FY2015**				1,169,251	1,169,251
**FY2016**	224,943		224,943	1,037,104	1,274,718
**FY2017**	234,026		234,026	533,650	780,774
**FY2018**	255,651		255,651	1,173,525	1,443,025
**FY2019**	264,303		264,303	1,243,032	1,522,315
**FY2020**	269,605		269,605	1,301,868	1,586,941
**FY2021**	302,075		302,075	1,529,686	1,849,203
**FY2022**	316,344	250,083	316,344	1,544,123	2,128,914
TOTAL	**1,866,947**	**250,083**	**1,866,947**	**9,532,239**	11,755,141

**Figure 1:**
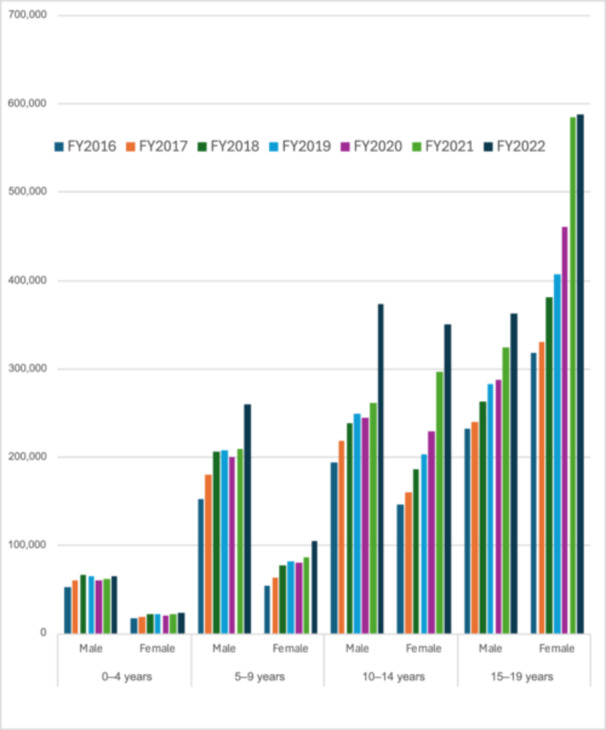
This figure illustrates the annual number of outpatient psychotherapy claims under specialized management fees, categorized by age group and sex, from fiscal year (FY) 2016 to FY2022.

Figure 1 illustrates the annual claims by age and sex. In the 15–19‐year‐old age group, claims from female patients increased from 318,174 in FY2016 to 587,888 in FY2022 (+85%), while claims from male patients increased from 231,953 to 362,226 (+56%). In the 10–14‐year‐old age group, claims nearly doubled for both male (from 194,008 to 373,898) and female (from 146,299 to 350,554) patients. In the 0–4‐year‐old age group, modest increases were observed in both sexes.

**Figure 2:**
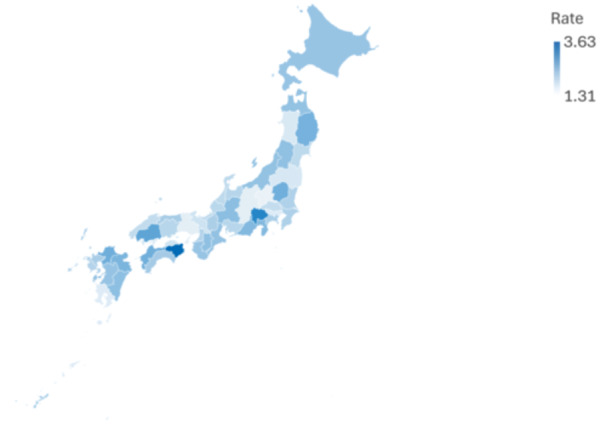
This figure shows the ratio of outpatient psychotherapy claims in fiscal year (FY) 2022 compared to FY2015 by prefecture. Most regions more than doubled their claims, with some (e.g., Tokushima and Yamanashi) showing more than a threefold increase, highlighting significant geographic variation in uptake.

The national average number of outpatient psychotherapy claims per 10,000 youth rose significantly from 521.0 in FY2015 to 1034.5 in FY2022 (*p* = 0.00042, *R*² = 0.891). However, regional disparities, as measured using the minimum‐to‐maximum ratio across prefectures, showed no significant changes (*p* = 0.204; Figure 2).

The effect sizes were largest among adolescent girls aged 10–14 years (+22.7%, *p* = 0.0005) and 15–19 years (+15.7%, *p* = 0.0004), followed by boys aged 15–19 years (+8.96%, *p* = 0.0002). All groups showed statistically significant increases except boys aged 0–4 years (*p* = 0.17).

### Adoption of the “specialized management” fee (under 16) by prefecture

In FY2016, 17 prefectures (36.17%) had not yet adopted the “specialized management” fee for patients under 16 years (Table 3). This number briefly increased to 18 in FY2017 (38.30%) but gradually decreased to 15 in 2018, 14 in 2019, 8 in 2020, and to just 3 (6.38%) in FY2022. As of FY2022, 93.62% of prefectures had implemented the fee.

**Table 3:** Number of calculations in FY2022 compared with that in FY2015 by prefecture.

FY	Hokkaido	Aomori	Iwate	Miyagi	Akita	Yamagata	Fukushima	Ibaraki	Tochigi	Gunma	Saitama	Chiba	Tokyo	Kanagawa	Niigata
FY2022	1217.96	861.78	776.99	712.75	451.11	1766.47	1019.55	686.79	757.95	821.55	659.53	853.12	1459.36	1085.37	1075.83
FY2021	941.74	764.07	626.44	754.70	459.36	1339.50	911.44	640.95	779.52	827.75	678.45	759.03	1283.10	943.00	905.30
FY2020	824.42	672.01	532.97	583.20	346.82	1150.35	777.36	578.82	654.66	710.78	537.57	635.38	1050.50	799.73	724.45
FY2019	757.64	573.14	426.68	517.35	330.22	1114.38	758.97	498.51	461.84	670.16	519.43	573.72	1062.70	788.21	702.15
FY2018	737.70	547.58	379.95	509.67	291.21	1016.18	727.81	422.66	354.25	682.48	482.44	516.05	1007.55	733.64	719.41
FY2017	753.50	512.84	443.73	473.62	250.52	901.35	717.75	390.31	308.52	613.44	452.55	488.09	951.77	658.53	730.69
FY2016	629.53	462.64	432.56	427.52	293.28	882.70	678.87	360.01	321.98	544.26	402.16	456.69	933.11	602.43	645.83
FY2015	552.22	383.40	310.68	378.28	275.53	775.31	618.25	342.78	296.43	548.91	362.54	424.08	901.64	529.23	479.41

FY	Toyama	Ishikawa	Fukui	Yamanashi	Nagano	Gifu	Shizuoka	Aichi	Mie	Shiga	Kyoto	Osaka	Hyogo	Nara	Wakayama	Tottori
FY2022	727.23	763.17	1188.60	705.54	847.39	1002.56	1537.45	1281.52	1058.99	642.69	817.47	1026.40	723.30	936.80	602.12	990.27
FY2021	640.45	728.78	1332.04	664.00	788.39	867.98	1051.61	1040.43	723.55	563.34	739.47	837.55	628.98	850.36	504.12	1013.01
FY2020	502.68	603.23	1055.03	523.18	673.95	772.82	924.12	907.10	684.83	456.88	625.69	730.66	554.45	710.83	507.00	1083.42
FY2019	483.52	541.36	982.62	478.66	642.66	591.97	891.00	881.46	655.21	411.57	644.37	725.75	545.08	710.70	438.74	593.55
FY2018	495.31	465.10	809.66	479.72	593.47	478.05	888.03	854.72	622.55	368.12	618.44	697.87	524.38	679.66	327.25	509.65
FY2017	481.00	425.11	475.46	408.86	575.97	533.50	801.52	783.06	579.31	372.57	568.95	627.19	508.32	576.02	311.96	422.58
FY2016	447.20	425.15	555.65	311.06	606.58	469.41	768.77	716.43	553.66	377.42	490.53	556.11	474.50	477.98	265.49	427.52
FY2015	373.26	403.58	623.89	223.76	543.94	431.44	641.95	661.36	469.45	315.22	468.63	517.81	468.43	410.55	272.63	474.29

FY	Shimane	Okayama	Hiroshima	Yamaguchi	Tokushima	Kagawa	Ehime	Kochi	Fukuoka	Saga	Nagasaki	Kumamoto	Oita	Miyazaki	Kagoshima	Okinawa
FY2022	936.96	1550.85	1183.57	631.62	1190.54	1074.05	922.35	681.52	1280.33	1564.23	881.89	1157.30	774.24	967.65	719.03	779.32
FY2021	859.93	1103.02	950.68	642.56	1010.78	938.56	857.71	523.75	998.37	1230.41	839.19	1044.13	543.81	629.59	750.20	678.46
FY2020	674.93	991.92	853.42	484.19	647.43	825.15	664.68	451.87	776.78	906.56	686.32	761.80	580.74	558.18	646.14	589.19
FY2019	689.98	1003.61	793.26	436.23	594.13	825.06	545.05	386.97	751.40	899.71	622.67	754.97	367.23	473.97	544.89	487.95
FY2018	552.75	992.66	566.19	399.24	561.19	807.68	481.25	372.93	722.66	831.60	539.32	700.98	371.64	498.62	522.29	443.43
FY2017	505.11	977.85	541.57	436.63	460.28	883.09	433.31	358.62	575.86	841.99	538.76	640.42	388.36	533.29	492.02	383.90
FY2016	501.34	980.46	501.14	486.75	366.64	870.98	400.54	376.06	541.96	845.31	481.57	565.67	314.77	398.38	471.56	402.59
FY2015	510.52	798.61	437.70	481.60	328.04	625.00	361.51	322.67	497.59	757.46	446.26	519.53	313.78	418.88	449.23	382.80

### Geographic trends and inter‐prefectural disparities

Outpatient psychotherapy claims per 10,000 youth more than doubled in most prefectures. For example:

- **Hokkaido**: 552.22 → 1217.96 (2.21×)
- **Kanagawa**: 529.23 → 1085.37 (2.05×)
- **Tokyo**: 901.64 → 1459.36 (1.62×)
- **Tokushima**: 3.63× increase
- **Yamanashi**: 3.15× increase
- **Kochi**: 2.11× increase.

Furthermore, the national averages nearly doubled (521.0 → 1034.5). The maximum claim rate rose from 901.64 to 1766.47, while the minimum increased from 223.76 to 451.11 (Table 4). The minimum‐to‐maximum ratio improved only slightly—from 4.03 to 3.92—indicating persistent inter‐prefectural disparities.

**Table 4:** Number of insurance points calculated per 100,000 population.

	Total of all prefectures	Maximum number of prefectures	Minimum number of prefectures	Minimum‐to‐maximum ratio
FY2015	52.1	90.16	22.38	4.03
FY2016	57.1.8	98.05	26.55	3.69
FY2017	61.48	97.79	25.05	3.90
FY2018	65.99	101.62	29.12	3.49
FY2019	70.4.6	111.44	33.02	3.37
FY2020	71.4.1	115.0.4	34.68	3.32
FY2021	88.29	133.95	45.94	2.92
FY2022	103.45	176.65	45.11	3.92
Multipliers for FY2022 compared to FY2015	1.99	3.63	1.31	2.77

## DISCUSSION

This study provides evidence that the MHLW insurance reforms increased utilization of outpatient psychiatric services billed under the new reimbursement categories. Because psychiatric outpatient charges from NDB Open Data do not include pediatric, developmental consultation, or general practice settings, our findings reflect only one segment of child and adolescent mental health care utilization in Japan. Between FY2015 and FY2022, claims for these services increased by 68%, demonstrating a growing demand and widespread institutional adoption of the revised reimbursement system. Furthermore, as an observational study, this analysis cannot establish causality between reimbursement reforms and increased utilization.

However, the aggregated nature of NDB Open Data means we cannot assess treatment quality, provider expertise, or therapeutic content; increased claim counts may not equate to improved clinical outcomes or consistent care quality across settings. Moreover, this finding indicated that adolescent girls showed significantly higher increases in service utilization compared to boys. Outpatient registry data from outpatient of the Japanese Council of Child and Adolescent Mental Institution indicate that preschool and early elementary school children are more likely to be boys and are mainly neurodevelopmental. While upper elementary and middle school children are more likely to be girls than boys, and mood and anxiety disorders increase.^11^ In other words, the high number of psychiatric visits among adolescent girls is assumed to be due to the absolute number, not the utilization of services.

The restructuring of reimbursement categories in FY2022 enabled institutions to align billing practices with patient characteristics and treatment intensity, which likely encouraged broader utilization of psychotherapeutic services. By FY2022, more than 93% of prefectures had implemented the “specialized management” fee, up from 64% in FY2016. This extensive adoption indicates progress toward the standardization of psychiatric service access nationwide.

The most substantial increases in service utilization were observed among adolescent girls, followed by boys in the same age group. Notably, even the youngest age group (0–4 years) showed gradual increases in claims, suggesting greater attention to early detection and intervention. These trends are particularly significant given the backdrop of a declining young population, implying that per capita access to services has expanded considerably.

Municipal programs that offer free medical care for children under 18 years likely contributed to the increase in access. The combination of national financial incentives and local subsidies may also have created a supportive environment for service expansion without imposing additional financial burdens on families.

Nevertheless, regional disparities remain. While prefectures such as Tokushima and Yamanashi experienced exceptional increases in claims, those of other regions were more modest. Because the NDB Open Data include only insurance claims, they do not capture local administrative initiatives—such as municipal outreach programs, expanded subsidies, or school‐based mental health services—that likely influence service uptake. For example, the 2024 Japan Child Mental Health Professional Shortage Areas index demonstrates that Tokushima Prefecture has substantially expanded its child and adolescent psychiatry workforce—by increasing the number of board‐certified specialists and specialized facilities—thereby reflecting real improvements in resource availability beyond billing data.^12^ These findings underscore the need for complementary qualitative and administrative data to fully understand the drivers of regional success.

From an implementation science perspective, the observed regional variations reflect differences in adoption (the extent to which prefectures took up the new reimbursement categories), fidelity (the degree to which billing practices aligned with the intended policy design), and contextual factors, such as local leadership, stakeholder engagement, and infrastructure readiness. Prefectures with strong inter‐sectoral coordination and robust provider networks achieved higher adoption and sustained fidelity, whereas areas with limited stakeholder engagement or resource constraints faced greater implementation challenges.

While our findings support the effectiveness of financial incentives in increasing outpatient psychiatric service utilization, fee schedule reforms should be complemented by investments in workforce training, specialist certification, and infrastructure improvements to ensure that increased access translates into high‐quality, sustainable care.

### Limitations

This study has several limitations. First, the NDB Open Data are aggregated and lack individual‐level clinical information. Therefore, increased claim counts may not reflect improvements in clinical outcomes or consistent care quality. These NDB Open data were limited to annual aggregated claims counts; we were not able to examine individual session information, such as actual session duration or therapeutic intensity.

Second, provider qualifications are not available from the database; thus, no indications can be obtained of whether services were delivered by board‐certified child and adolescent psychiatrists. This limits the assessment of the impact of specialist involvement on service utilization.

Third, due to a 2‐year delay in the release of data, timely policy evaluation will have been constrained. Furthermore, only FY2022 includes population data by age and prefecture, requiring the use of claim counts rather than unique patient data across the preceding fiscal years.

Fourth, we were able to analyze the opening of the NDB starting in 2014. Our dataset covers FY2014–2022; reforms of psychiatric treatment for children initiated in 2012 are not included, potentially underestimating the underlying policy impact. Lastly, because this was an observational study relying on administrative data, causal relationships could not be established. Regional differences in service utilization may also indicate unmeasured factors, such as local healthcare capacity, municipal policies, or socioeconomic conditions.

### Conclusion

The introduction of additional reimbursement points by the MHLW has successfully expanded outpatient psychiatric care for children and adolescents in Japan. Service utilization nearly doubled between FY2015 and FY2022, with a significant majority of prefectures adopting the policy. The strongest uptake was observed in regions such as Tokushima and Yamanashi, indicating that local factors may amplify policy effects.

These findings demonstrate the importance of the combined impact of sustained national policy support and local implementation efforts. Financial incentives, when coupled with municipal subsidies that reduce out‐of‐pocket costs, may create an effective framework for improving access to care.

Future efforts should focus on enhancing the quality of care, increasing the number of trained specialists, and addressing persistent regional disparities. A more equitable and robust child and adolescent psychiatric care system will require not only financial commitment but also ongoing evaluation and support for local health infrastructure.

## AUTHOR CONTRIBUTIONS

Masahide Usami designed this study and wrote this manuscript. Yoshinori Sasaki, Masahiro Ishida, Saori Inoue, Masaya Ito, Katsunaka Mikami, Noa Tsujii, and Naoko Satake discussed this review.

## CONFLICT OF INTEREST STATEMENT

The authors declare no conflicts of interest.

## ETHICS APPROVAL STATEMENT

The NDB Open Data are publicly available and anonymized. No ethical approval or patient consent was required under the Japanese guidelines.

## PATIENT CONSENT STATEMENT

N/A.

## CLINICAL TRIAL REGISTRATION

N/A.

## Data Availability

N/A.
